# Association of dietary inflammatory index with C-reactive protein and interleukin-6 in women with and without polycystic ovarian syndrome

**DOI:** 10.1038/s41598-024-53958-5

**Published:** 2024-02-17

**Authors:** Khadijeh Azarbayjani, Shahideh Jahanian Sadatmahalleh, Azadeh Mottaghi, Maliheh Nasiri

**Affiliations:** 1 Department of Reproductive Health and Midwifery, Faculty of Medical Sciences, Tarbiat Modares University, Tehran, Iran; 2 Research Center for Prevention of Cardiovascular Diseases, Institute of Endocrinology and Metabolism, Iran University of Medical Sciences, Tehran, Iran; 3 Department of Basic Sciences, Faculty of Nursing and Midwifery, Shahid Beheshti University of Medical Sciences, Tehran, Iran

**Keywords:** Diseases, Endocrinology, Medical research

## Abstract

Considering that interventions related to lifestyle, especially nutrition have been proposed as the first line of prevention and treatment of polycystic ovarian syndrome (PCOS), and regarding the proven relationship between PCOS and inflammation, the present study was designed to find out the possible association of Diet Inflammatory Index (DII) with the inflammatory markers like C-reactive protein (CRP) and Interleukin-6 (IL-6), and compare the obtained results in PCOS and non-PCOS women. This case–control study was conducted on 45 PCOS and 40 non-PCOS women. Food intake and DII were measured using a 147-item Food Frequency Questionnaire. All participants were tested for the serum levels of IL-6 and CRP. Finally, the obtained results were compared between the two groups of PCOS and non-PCOS women. Significant differences were observed between the two groups in terms of age, menstrual status and number of pregnancies (*P* < 0.05). Comparison of DII values showed no significant difference between the two groups of women (*P* = 0.68), but IL-6 was significantly higher in the PCOS group than in the control group (4.94 ± 1.97 vs. 3.48 ± 1.77, *P* < 0.001). Also in terms of CRP, no significant difference was observed between the two groups (*P* > 0.05). The difference of DII between the case and control groups were not significant and Pearson's correlation test did not show a significant correlation between DII and IL-6 (*P*˃0.05). This result can be due to the influence of several factors affecting the determination of DII such as education level, health status, physical activity level, age, and calorie intake. It seems that diet, especially consumption of more carbohydrates plays a role in causing chronic inflammation, as well as the occurrence and exacerbation of PCOS.

## Introduction

Polycystic Ovarian Syndrome (PCOS) is the most prevalent female endocrine disorder, which is characterized by various collections of symptoms like elevated androgen level, menstrual abnormality and morphological manifestations of polycystic ovaries in reproductive-aged women^1^. Approximately 4–20% of women of childbearing age are affected by this disorder and its associated complications^2^. PCOS is a lifelong disorder in women, and its metabolic and reproductive effects are well known in women's lives^3^. PCOS is a condition with chronic mild inflammation, which is associated with an increase in some inflammatory markers such as C-reactive protein (CRP)^4^. Diet plays a major role in regulating chronic inflammation, and inflammation caused by diet is the basis for insulin resistance and Hyperandrogenism, which are the main components of PCOS^5^. It has been shown that some food ingredients like glucose can cause chronic inflammation through oxidative stress^5,6^. Dietary Inflammatory Index (DII), designed and developed by Shivappa et al., is a quantitative scale for classify individuals’ diet according to their inflammatory potential. DII assays the association between the dietary components with inflammatory markers like IL-1β, IL-4, IL-6, IL-10, TNF-α, and CRP^7^. Some foods such as garlic, curcumin and fibers have anti-inflammatory properties and a lower DII. A pro-inflammatory diet, on the other hand, contains a lot of whole-fat foods, refined cereals, fats, fruit juices, red meat, processed meat, sugar-sweetened beverages, sweets, sugar, and honey^8^. Recent studies have reported that higher DII values are associated with a higher risk of PCOS^9^. Interventions related to lifestyle changes, including nutrition, exercise and weight loss are the first line of treatment for PCOS women^10^. Changes in the composition of diet for the management, treatment and even prevention of this disorder have recently gained much research attention. Furthermore, making changes in the composition of diet causes a decrease in androgen hormones that lead to the emergence of clinical symptoms of PCOS^11^. Therefore, finding the right diet is important, especially for thin women with PCOS. The present study aimed to investigate the possible association of DII with the inflammatory markers like CRP and Interleukin-6 (IL-6), and compare the obtained results in PCOS and non-PCOS women.

## Methods

### Participants

**Figure 1:**
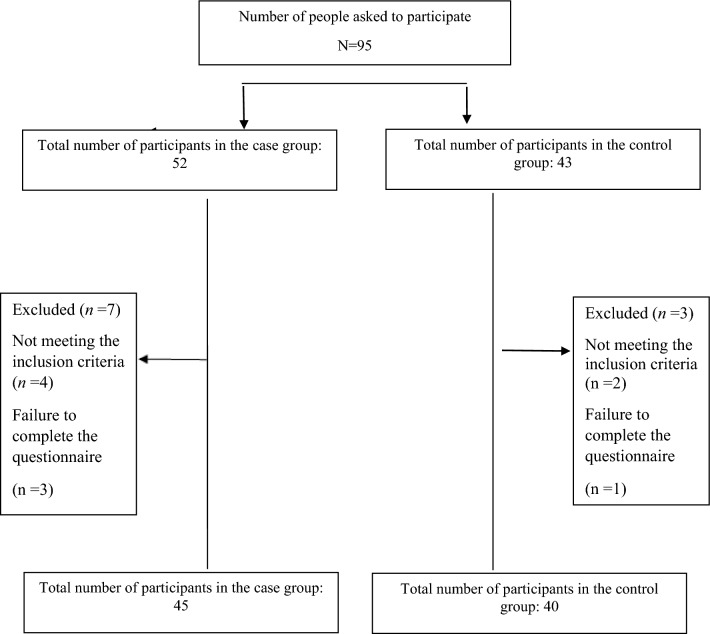
Flow chart of the study.

This case–control study was conducted on a total of 85 Iranian women in two groups: a case group consisting of women with PCOS (n = 45) and a control group of 40 healthy women with the aim of investigating the possible association of DII with the inflammatory markers like CRP and IL-6, and compare the obtained results in PCOS women and healthy control group (non PCOS women). Prior to commencing the study, ethical clearance was sought from The Ethics Committee of Tarbiat Modares University of Medical Sciences (IR.MODARES.REC.1399.177). A random sample of women was recruited from those referring to one of the Obstetrics and Gynecology clinics in Robat Karim City, located in Tehran Province between May 2021 and February 2022. The subjects were selected with non-probability convince sampling method. They were in the age group of 18–45 years and had a BMI less than 40 kg∕m^2^. The participants were divided into two groups according to having and not having PCOS based on the Rotterdam criteria and with a physician diagnosis, or having a previous history of PCOS diagnosis based on medical records. Due to lack of similar studies, at first, 20 PCOS women and 20 healthy women without PCOS were selected to conduct a pilot study. The calculation of an adequate sample size was done based on the results of the unpublished pilot study and using the following formula:

$$n \ge 2\frac{{\left( {z_{\alpha /2} + z_{\beta } } \right)^{2} \sigma^{2} }}{{\left( {\mu_{1} - \mu_{2} } \right)^{2} }}$$

where,

$$\begin{aligned} \alpha & = 0.05 \Rightarrow z_{\alpha /2} = 1.96 \\ \beta & = 0.10 \Rightarrow z_{\beta } = 1.28 \\ \end{aligned}$$

$$1 - \beta = 0.90$$

$$n = 2\left( {1.96 + 1.28} \right)^{2} \left( {\frac{1}{0.73}} \right)^{2} = 40.$$

where, α is the probability of type 1 error, and β is the probability of type 2 error, which is 0.1 with 90% power. The observed effect size based on the level of IL-6 in a pilot sample of 40 women (20 women in each group) was equal to 0.73, (μ1–μ2 = 0.73 .(Considering the obtained 95% confidence interval, 90% power and 0.73 effect size in the pilot sample and substituting these numbers in the above formula, the minimum sample size in each group was determined to be 40 women. Figure 1 shows the flow chart of the study.

Criteria for selecting the subjects were as follows: women of reproductive age, not having fever and cold symptoms, not suffering from chronic diseases (e.g., diabetes, atherosclerosis, high blood pressure, liver and kidney diseases, rheumatic diseases, etc.) according to medical records and individual reports, absence of other inflammatory diseases like endometriosis and myoma, no pregnancy , no breastfeeding, not taking Anti-Hypertensive and Anti-Diabetics drugs and drugs that affect appetite, and having PCOS according to the Rotterdam criteria in the case group. The women unwilling to continue the research or having energy intake more than 4200 or less than 800 were excluded from the study. Before data collection, the participants received an explanation of the project. After obtaining written informed consent from the all participants in both groups, a questionnaire of demographic information (age, marital status, employment status, education level, height and weight, history of chronic diseases, history of drug use, and fertility and menstruation information was completed. Also all participants were subjected to blood pressure, height and weight measurement and Body Mass Index (BMI) calculation by the researcher. The visual analogue scale was used to calculate the severity of dysmenorrhea. The participants were asked to report their related pain score. This scale has a range from 0 to 10, which means the lowest and the highest pain score, respectively^12^. To evaluate the physical activity, all sports activities reported by the participant during the week based on hours are multiplied by the number of days of exercise and the amount of exercise is calculated based on hours per week.

### Dietary assessment

The participants’ nutritional intake was obtained through a 147-item Food Frequency Questionnaire (FFQ) whose validity and reliability were previously evaluated^13^. After receiving the necessary training and consultations in the field of completing and interpreting the FFQ from the nutritionist member of the research team, it was completed for all participants in the two groups by the researcher. The information obtained from the questionnaire was entered into the IV Nutritionist software to obtain the exact intake of energy and micronutrients. The participants were asked to report the frequency of consumption of each food item during the last year according to the size of certain portions. The frequency of consumption was converted to daily intake (grams per day). Finally, by summing up the values of each nutrient, the total dietary intake of that nutrient was calculated.

### Calculation of DII

The data related to 31 food items (available in the existing studies), including vitamin B12, vitamin B6, vitamin A, vitamin C, vitamin D beta-carotene, folic acid, fiber, fats, energy, cholesterol, carbohydrates, caffeine, iron, magnesium, niacin, protein, riboflavin, selenium, thiamin, zinc, trans fatty acids, saturated fatty acids (SFAs), mono-unsaturated fatty acids (MUFAs), poly-unsaturated fatty acids (PUFAs), garlic, onion, saffron, turmeric, black tea and pepper were used in order to evaluate the DII. The food ingredients were given score + 1, −1, or 0 according to their role (positive, negative or neutral) in inflammation, respectively. To calculate the DII, the daily intake of each food item was subtracted from the world standard average and divided by the standard deviation. These values were then converted into a central percentile score. Each percentile score was multiplied by 2, and 1 is subtracted. The central percentile value for each food parameter was then multiplied by the corresponding "Overall Food Parameter Specific Inflammatory Effect Score"^14^.

### Measurement of IL-6 and CRP

After 12–14 h of fasting, 5 cc of venous blood samples was taken from all participants to measure their inflammatory markers. Then the samples were centrifuged and the resulting plasmas were stored in a freezer at −20 °C until the tests were performed. In this study, the human IL-6 ELISA kit produced by the Zell Bio Company (Germany) the measurement unit of pg/ml was used. The basis of this method is an immunoassay based on sandwich ELISA. Moreover, CRP was measured using the latex agglutination method; values less than 40 mg/liter were considered as negative and values above 40 mg/liter were considered as positive.

### Statistical analysis

Statistical analysis was performed using the SPSS software (ver. 20). Independent t-test was used in order to compare the two groups in terms of quantitative variables such as age and BMI. Qualitative variables were compared using the Chi-square test. A *P* < 0.05 level was chosen for statistical significance. Considering that the two groups were not matched in terms of variables like age and number of children, the analysis of covariance test was used to compare the two groups in terms of DII and other food parameters, as well as the IL-6.

### Ethics approval and consent to participate

Ethical approval for the present study was obtained by The Ethics Committee of Tarbiat Modares University of Medical Sciences (IR.MODARES.REC.1399.177)**.** All study protocols were in accordance with the ethical standards of the Regional Research Committee, as well as the Declaration of Helsinki 1964 and its later amendments. After explaining the research purposes, informed written consent and verbal assent were obtained from all participants. They were also informed that their participation was voluntary, confidential, and anonymous and that they had the right to withdraw from the research at any time.

## Results

The present case–control study was conducted on 45 women with PCOS (case group) and 40 non-PCOS women (control group). After the interview, 10 participants (10.5%) were excluded from the study due to not meeting the inclusion criteria (unwilling to continue participating in the study and participating in laboratory investigations, finding out about pregnancy between completing the questionnaire and performing laboratory tests and failure to complete the questionnaire) (Fig. 1). Mean ± standard deviation was used to describe quantitative variables, and number and percentage were used for qualitative variables. Table 1 presents a comparative analysis of demographic and fertility characteristics in the two groups. The mean age of women in the case and control groups was 27.93 ± 6.64 and 33.02 ± 7.19 years, respectively. A significant difference was observed between the two groups in terms of age, number of pregnancies, and menstrual status (*P*˂0.05). As shown in Table 1, there was no significant difference between the two groups in terms of other demographic variables such as BMI, education level, occupation status, marital status, income, housing, smoking, alcohol consumption and amount of exercise (*P*˃0.05). Comparison of fertility variables, including menarche age, menstrual pain score, duration of bleeding, history of infertility, type of delivery and contraceptive method showed no statistical difference between the two groups (*P*˃0.05).

**Table 1:** Examining the demographic and fertility characteristics in the case and control groups.

Variable	Case group N = 45 Mean (SD)	Control group N = 40 Mean (SD)	*P*-value
Age (year)*	27.93 (6.64)	33.02 (7.19)	0.001
BMI*	25.24 (3.93)	25.85 (5.07)	0.53
Menarche age*	13.15 (1.46)	13.57 (1.51)	0.19
Score of dysmenorrhea*	5.93 (2.59)	5.70 (2.94)	0.15
Duration of bleeding*	6.24 (1.62)	5.62 (1.89)	0.28
Number of pregnancy*	0.84 (1.12)	1.70 (2.00)	0.04

	Number (%)	Number (%)	*P*-value
Menstrual status**			
Regular	20 (44.4)	29 (72.5)	0.009
Irregular	25 (55.6)	11 (27.5)	
Interval of menstruation**			
˂21	1 (12.2)	1 (12.5)	0.07
21–35	29 (64.4)	34 (85)	
> 35	15 (33.3)	5 (12.5)	
Dysmenorrhea**			
Yes	31 (68.9)	22 (55)	0.18
No	14 (31.1)	18 (45)	
Menorrhagia**			
Yes	14 (31.1)	9 (22.5)	0.37
No	31 (68.9)	31 (77.5)	
History of infertility**			
No history of infertility	42 (93.3)	38 (95)	0.38
Primary	3 (6.7)	1 (2.5)	
Secondary	0 (0)	1 (2.5)	
Type of delivery**			
No history of delivery	25 (55.6)	20 (50)	0.83
NVD	12 (26.7)	13 (32.5)	
CS	8 (17.8)	7 (17.5)	
Contraception method**			
OCP	3 (6.8)	1 (2.5)	0.33
IUD	1 (2.3)	2 (5)	
Other	16 (36.4)	21 (52.5)	
None	24 (54.5)	16 (40)	
Education (year)**			
≤ 12	35 (77.8)	28 (70)	0.27
˂12	10 (22.2)	12 (30)	
Occupation**			
Housewife	29 (64.4)	18 (45)	0.12
Employed	16 (35.6)	22 (55)	
Marital status**			
Single	17 (37.8)	10 (25)	0.26
Married	27 (60)	30 (75)	
Widow	1 (2.2)	0 (0)	
Income )million tomans(**			
˃2.5	15 (33.3)	6 (15)	0.10
2.5–5	19 (42.2)	18 (45)	
> 5	11 (24.4)	16 (40)	
Housing**			
Private	32 (71.1)	27 (67.5)	0.71
Rental	13 (28.9)	13 (32.5)	
Smoking**			
Yes	4 (8.9)	4 (10)	0.86
No	41 (91.1)	36 (90)	
Alcohol consumption **			
Yes	3 (6.66)	3 (7.5)	0.54
No	42 (93.33)	37 (92.5)	
Exercise(hours per week)**			
˃1	32 (71.2)	31 (77.5)	0.76
3–1	11 (24.4)	8 (20)	
˂3	2 (4.4)	1 (2.5)	

*BMI* body mass index, *NVD* natural vaginal delivery, *CS* Caesarean section, *OCP* oral contraceptive pills, *IUD* intra uterine device.

*Values are given as Mean ± SD using the independent *t*-test.

**Values are given as number and percentage using the Chi-square test.

As shown in Table 2, no significant difference was observed between the two groups in terms of calorie intake, dietary macronutrients and some micronutrients (*P*˃0.05), but there was a significant difference in daily food intake (in grams) between the two groups, which was higher in the control group (*P*˂0.05).

**Table 2:** Examination of dietary intake and dietary macronutrients and micronutrients in the case and control groups.

Variable	Case group (N = 45) Mean (SD)	Control group (N = 40) Mean (SD)	*P*-value*
Daily food intake (in grams)	2094.72 (691.55)	2546.83 (539.66)	0.02
Daily calorie intake (in kilocalories)	2324.42 (719.79)	2687.70 (709.53)	0.11
Daily intake of protein	80.74 (27.47)	96.46 (28.57)	0.77
Daily intake of carbohydrates	342.39 (115.07)	387.16 (109.69)	0.26
Daily intake of total fat	77.03 (28.26)	91.92 (28.63)	0.74
Daily intake of fiber	57.97 (26.75)	68.17 (27.00)	0.20
Daily intake of cholesterol	209.32 (96.12)	247.01 (101.57)	0.27
Daily intake of beta-carotene	3425.18 (2204.14)	3862.22 (2077.04)	0.96
Daily intake of Vitamin C	121.11 (59.07)	141.97 (56.07)	0.28
Daily intake of Vitamin E	11.55 (6.16)	13.17 (6.65)	0.41
Daily intake of zinc	11.74 (4.5)	14.20 (4.3)	0.11
Daily intake of glucose	14.7 (6.49)	16.50 (5.95)	0.60
Daily intake of selenium	129.50 (64.26)	148.98 (54.64)	0.32

*Values are given as Mean ± SD using the analysis covariance test.

Table 3 compares DII values in the case and control groups. As shown, no significant difference was observed between the two groups in terms of DII (*P*˃ 0.05).

**Table 3:** Comparison of DII values in the case and control groups.

Variable	Case group	Control group	*P*-value*
	(N = 45) Mean (SD)	(N = 40) Mean (SD)	
DII	4.11 (1.51)	4.36 (0.44)	0.68

*Values are given as Mean ± SD using the analysis covariance test. DII: Diet Inflammatory Index.

As displayed in Table 4, the serum level of IL-6 was significantly higher in the case group than in the control group (*P*˂0.05); however, the difference between the two groups was not significant in terms of CRP.

**Table 4:** Comparison of the serum levels of IL-6 and CRP between the case and control groups.

Variable	Case group (N = 45) Mean (SD)	Control group (N = 40) Mean (SD)	P-value
IL-6*	4.94 (1.97)	3.48 (1.77)	<0.001
CRP**			
Positive	2 (4.5)	1 (2.5)	0.62
Negative	43 (95.5)	39 (97.5)	

*IL-6* interleukin-6, *CRP* C-reactive protein.

*Values are given as Mean ± SD using the analysis covariance test.

**Values are given as number and percentage using the Chi -square test.

Pearson’s correlation test was used to investigate the relationship between DII and IL-6. As shown in Table 5 there is no significant correlation between these two parameters (*P*˃ 0.05).

**Table 5:** Investigating the correlation between IL-6 and DII.

Variable	IL6
DII	0.96*

*DII* diet inflammatory index, *IL-6* Interleukin-6.

*Values are given by Pearson’s correlation test.

## Discussion

**Figure 2:**
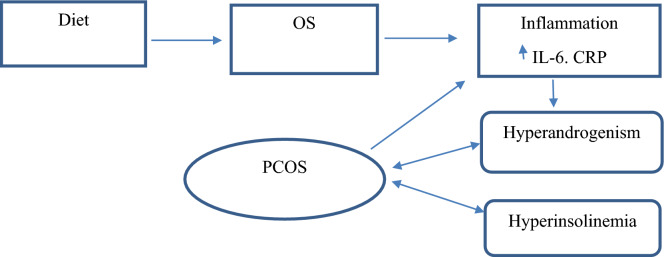
The relationship between diet, inflammation and PCOS.

Chronic low-grade inflammation has contributed in the pathogenesis of PCOS. Diet, as one of the main components of a healthy lifestyle, can moderate the inflammatory condition, especially in women with PCOS (Fig. 2). This study was designed to investigate the possible association of DII with the inflammatory markers like CRP and IL-6, and compare the obtained results in women with PCOS and healthy control group (non-PCOS). Various studies have assessed the role of diet in women with PCOS; however, to the best of our knowledge, no study has assessed the association between DII and inflammatory markers in PCOS and non-PCOS women. According to the obtained results, the amount of daily food intake was significantly higher in the non-PCOS women comparing to the PCOS women; however, the two groups of women did not have significant differences in the daily intake of calorie, protein, carbohydrate and total fat; this finding is consistent with the results of several other studies. Wright et al.^15^ reported that no significant difference was observed between the case and control groups in food intake and physical activity. Also Alvarez et al.^16^ reported that there was no significant difference in daily food intake and the intake of macronutrients and micronutrients in the diet of PCOS and non-PCOS women but the intake of white bread (with a higher glycemic index) was significantly higher in the PCOS group. Contrarily, in the study of Barr et al.^17^, a higher amount of daily food intake was observed in women with PCOS when compared to healthy women. There are several explanations for these conflicting results:

A. Studies evaluating the diet and physical activity of PCOS and non-PCOS women have used broad definitions of PCOS, which makes interpretation and comparison of different results challenging, because the group of women with PCOS consists of several distinct clinical phenotypes. Most of these studies have used the Rotterdam criteria. This may lead to creation of various and heterogeneous PCOS phenotypes; there are hormonal and metabolic differences among these clinical phenotypes, which may act as a confounding factor. These results are in agreement with the findings of Graff et al.^18^, who reported certain differences in dietary intake among the clinical phenotypes of PCOS. As a matter of fact, women with mild phenotype of PCOS have different metabolic status and health risks compared to those suffering from more severe phenotypes of PCOS^19^. This fact pinpoints the need to make distinction between the different phenotypes of PCOS in order to accurately compare lifestyle habits, including nutrition between PCOS and non-PCOS women.
B. Energy balance is an important determinant of weight and has not been adequately addressed in the literature. To our knowledge, few studies have simultaneously assessed physical activity while examining diet in women with PCOS^20^. On the other hand, the amount of daily energy intake is an effective factor in determining DII and comparing it between the groups. In the present study, no significant difference was observed in daily energy intake between the two groups. Evidence suggests that depression or low self-esteem in PCOS women increases the risk of eating disorders and reduced physical activity^21^. Therefore, one of the factors that can affect the relationship between diet and PCOS is mental health status in women.

One unanticipated finding in the present study was that no significant difference was found between the case and control groups in terms of DII. Contrary to this finding, Zirak Sharkesh et al.^22^ found a higher DII score associated with a higher incidence of PCOS. Likewise, Whang et al.^23^ reported that higher DII score had a positive correlation with the risk of PCOS. In accordance with the present research results, Zheng et al.^24^ observed no significant difference in the daily food intake and eating habits of obese and infertile PCOS women and obese non-PCOS women. The possible explanation for this finding might be that high carbohydrate intake, low-grade inflammation and Hyperandrogenism interact to affect the pathophysiology of PCOS. In fact, higher carbohydrate consumption increases DII, and thus, the incidence of PCOS^25^ while, according to the present study results, the two groups did not differ in terms of carbohydrate intake. Gonzalez et al.^26,27^ outlined the key role of food ingredients such as glucose and SFAs in causing inflammation and production of androgens, even independent of obesity and insulin resistance. Based on the findings of Barrea et al.^28^, the Mediterranean diet has been proposed as an anti-inflammatory food pattern that includes complex carbohydrates, fiber and SFAs. They found that patients with PCOS had higher consumption of simple carbohydrates, total fat and fatty acids. In addition, testosterone levels in patients with PCOS had a significant negative relationship with the intake of protein and complex carbohydrates. Moreover, a significant positive relationship was observed between CRP levels and the intake of simple carbohydrates. Gonzalez et al.^29^ found an evidence of increase in TNFa and IL-6 in women with PCOS under both conditions of increased glucose consumption in vivo and exposure to glucose in vitro, which was associated with insulin resistance, showing that inflammation caused by diet in PCOS patients is probably related to glucose consumption and insulin resistance**.**

Diet is a key determinant of overweight and the relationship between diet and PCOS is influenced by geographical factors and various dietary patterns^30^. The current research results support evidence from previous observations that reported no significant relationship between various dietary patterns, including the Mediterranean diet and PCOS, whereas, according to some other research results, it was expected that the Mediterranean diet, which has an inverse relationship with DII, would have a significant relationship with PCOS^31^. In summary, one can conclude that a healthy diet with a sufficient ratio of complex to simple carbohydrates should be appropriate to end with weight loss and improve metabolic, hormonal and reproductive homeostasis in women with PCOS.

In the present study, a significant difference was observed in terms of IL-6 between the two groups of PCOS and non-PCOS women, which was higher in the PCOS women. This finding is in agreement with the findings of Artimani et al., who also reported that the level of IL-6 was higher in PCOS patients compared to non-PCOS women^32^. The study conducted by Zanganeh et al. showed that the serum levels of IL1α and IL1β in women with PCOS were higher, but IL17 levels in PCOS women were significantly lower than in the non-PCOS control group^33^. This finding is contrary to the findings of Mohlig et al. in which the hypothesis of the relationship between PCOS and chronic inflammation was rejected. They found that neither CRP nor IL-6 in obese and thin women with PCOS was different from their counterpart non-PCOS women^34^. IL-6 is a pro-inflammatory marker that plays an important role in the development of both acute and chronic inflammation response. Adipose tissue produces IL-6 related to glucose and lipid metabolism^35^. Recent studies have shown that IL-6 has a significant relationship with insulin resistance, which plays an important role in the pathogenesis of PCO. On the other hand, Hyperandrogenism and Hyperinsulinemia can aggravate the inflammatory condition and create a vicious cycle^36^. Obesity acts as a potential confounding factor of CRP increase, which can affect the process of inflammation and the level of inflammatory markers^37^. Studies that assess the relationship between PCOS and inflammation and inflammatory markers might have several limitations. Most of these studies have small sample size and only women with approved diagnosis of PCOS based on the Rotterdam criteria are included in the study, which is the reason for the diversity in the study population due to the inclusion of different PCO phenotypes. The effect of obesity, especially visceral fat on inflammation is well known, and the results of most of these studies are not adjusted for the effect of obesity and other diseases affecting inflammation.

Our findings demonstrated that there was no correlation between DII and IL-6. Similarly, Kotemori et al.^38^ did not observe a relationship between DII and the level of CRP. Also, in a study conducted in the USA, despite the high level of CRP, no significant relationship was observed between CRP concentration and DII^39^. Contrary to these results, some other studies in western societies have reported a positive and significant relationship between inflammatory markers and DII^40,41^. The diversity in habits and diet as well as the different spectrum of inflammatory status in different societies can be one of the reasons for reporting different results in these studies. Moreover, the drugs used by the subjects in some of these studies have been responsible for the positive relationship observed between DII and inflammatory markers^38^.

### Strengths and limitations of the study

We used the valid and reliable FFQ for Iranian population, which is a suitable tool for nutritional investigations and determining the DII. There were several limitations with this study. The sample size was small and phenotypic division was not done in the PCOS group; so, it is suggested that future studies be conducted with larger sample sizes and create a clear distinction between the different phenotypes of PCOS. It would have been better if we had evaluated variables such as mental health status of participants as well as metabolic parameters.

## Conclusions

The present research findings support the results of recent studies regarding the increase of inflammatory markers like IL-6 in women with PCOS though there was no correlation between IL-6 and DII. It seems that diet, especially carbohydrates and glucose is capable to trigger an inflammatory response, and thus, affect the incidence and severity of PCOS; however, as discussed earlier, this relationship is influenced by various factors such as clinical phenotypes of PCOS, level of physical activity, level of education, amount of energy intake and mental health status. Therefore, in order to better examine and provide a suitable diet according to the conditions of each person, these factors should be taken into consideration in future studies.

## Data Availability

The data sets used and analyzed for the current study are available upon reasonable request from the corresponding author, Dr. Shahideh Jahanian (shahideh.jahanian@modares.ac.ir).

## Abbreviations

PCOS: Polycystic ovary syndrome
PCO: Polycystic ovaries
CRP: C-reactive protein
DII: Diet Inflammatory Index
PI: Phytochemical Index
FFQ: Food Frequency Questionnaire
TNF: Tumor necrosis factor
BMI: Body Mass Index
OS: Oxidative stress
